# TB infection prevention and control experiences of South African nurses - a phenomenological study

**DOI:** 10.1186/1471-2458-11-262

**Published:** 2011-04-25

**Authors:** Dagmar Sissolak, Frederick Marais, Shaheen Mehtar

**Affiliations:** 1 Academic Unit for Infection Prevention and Control, Department of Interdisciplinary Health Sciences, Faculty of Health Sciences, University of Stellenbosch & Tygerberg Academic Hospital, Tygerberg Campus, 9th Floor, Tygerberg Hospital, Tygerberg 7505, Cape Town, South Africa

**Keywords:** tuberculosis, TB, infection prevention and control, nurses, wards, clinical practice, barriers, phenomenological approach

## Abstract

**Background:**

The tuberculosis (TB) epidemic in South Africa is characterised by one of the highest levels of TB/HIV co-infection and growing multidrug-resistant TB worldwide. Hospitals play a central role in the management of TB. We investigated nurses' experiences of factors influencing TB infection prevention and control (IPC) practices to identify risks associated with potential nosocomial transmission.

**Methods:**

The qualitative study employed a phenomenological approach, using semi-structured interviews with a quota sample of 20 nurses in a large tertiary academic hospital in Cape Town, South Africa. The data was subjected to thematic analysis.

**Results:**

Nurses expressed concerns about the possible risk of TB transmission to both patients and staff. Factors influencing TB-IPC, and increasing the potential risk of nosocomial transmission, emerged in interconnected overarching themes. Influences related to the healthcare system included suboptimal IPC provision such as the lack of isolation facilities and personal protective equipment, and the lack of a TB-IPC policy. Further influences included inadequate TB training for staff and patients, communication barriers owing to cultural and linguistic differences between staff and patients, the excessive workload of nurses, and a sense of duty of care. Influences related to wider contextual conditions included TB concerns and stigma, and the role of traditional healers. Influences related to patient behaviour included late uptake of hospital care owing to poverty and the use of traditional medicine, and poor adherence to IPC measures by patients, family members and carers.

**Conclusions:**

Several interconnected influences related to the healthcare system, wider contextual conditions and patient behavior could increase the potential risk of nosocomial TB transmission at hospital level. There is an urgent need for the implementation and evaluation of a comprehensive contextually appropriate TB IPC policy with the setting and auditing of standards for IPC provision and practice, adequate TB training for both staff and patients, and the establishment of a cross-cultural communication strategy, including rapid access to interpreters.

## Background

With a population of approximately 43 million, South Africa (SA) has one of the highest incidence rates of *Mycobacterium tuberculosis *(TB) in the world, with annual notification rates in some areas in the Western Cape exceeding 1000/100000 [1]. Moreover, SA has one of the world's largest human immunodeficiency virus (HIV) epidemics, an estimated 5.54 million people being infected [2]. Measures for TB-infection prevention and control (IPC) in SA remain the responsibility of individual healthcare facilities [3]. There is growing evidence that hospital transmission is a critical factor in epidemic HIV-associated TB [4,5]. IPC can reduce the risk of TB transmission even in settings with limited resources [6]. A study, published elsewhere, on the potential to transmit TB at our hospital confirmed the high incidence of TB and a substantial risk for transmission [7]. This situation intensifies the need for a comprehensive hospital-based IPC programme to prevent the transmission of TB.

Internationally, TB-IPC is based on a three-level hierarchy of controls, including administrative, environmental, and respiratory protection [8]. The magnitude of the local TB burden, exacerbated by limited financial and human resources at public healthcare facility level, challenges the applicability and impedes the implementation of international guidelines.

Limitations in effective TB control worldwide have caused a shift in perspective, it is no longer considered a mere technical bio-medical intervention [9]. This applies to IPC practices at both hospital and community level. Effective TB-IPC requires adherence to measures which should be regarded as a chain of responsibilities, involving healthcare staff and decision-makers, as well as patients, and society [10]. Several recent studies have looked at non-biological influences on TB control, from the point of view of the patient [11,12], community [13] and health care providers [14,15]. Nurses play a central TB-IPC role in detecting the disease, providing and coordinating appropriate treatment, and assuring emotional support [16], but it seems a neglected area of research in high TB burden countries. There is a lack of information concerning the realities faced by nurses in implementing TB-IPC measures. The absence of nurses' voices constrains the quality and quantity of human resources for TB control and care [17]. As a result, health systems in a number of countries are weak and ineffective in meeting the growing need for TB control services [17]. One study in SA reported nurses' lack of awareness of beliefs and attitudes about TB harboured by communities they serve, nor of their behaviour concerning illness [11].

This paper reports findings from a qualitative study seeking to explore factors influencing TB-IPC practices at hospital level from the experiences of ward nurses in order to identify risks associated with potential nosocomial transmission, and to emphasize the crucial role nurses play in TB control and care.

## Methods

### Setting

At the time of the study, the tertiary academic hospital in Cape Town, South Africa, contained 1291 beds, of those approximately 26% surgical, 20% intensive care unit (ICU) and emergency (both internal medicine and surgery), 12% obstetric/gynaecological, 16% internal medicine, 20% paediatrics and 6% for minor sub-specialities. There were 1,502 nurses employed, of whom about 75% were of coloured origin, 19% African, and 6% white. Exact figures were not available, but all nurses were conversant in English of whom approximately 80% spoke Afrikaans and some Xhosa as their first language.

### Design

The qualitative data was collected during one-to-one in-depth interviews with a semi-structured interview guide (Table 1) to explore ward nurses' experiences of factors influencing TB-IPC practices, using phenomenological approach. The purpose of phenomenological research is to describe what people experience in regard to certain phenomena, as well as how they interpret the experiences or what meaning the experiences hold for them [18]. Therefore, phenomenology is an approach that concentrates on a subject's experience rather than on the person as a subject or object [18].

**Table 1:** Interview guide.

1. Do you usually deal with diagnosed TB patients at your workplace?
2. What protective device are in place and used on the wards:
• Surgical masks
• N95-respirators
• Gloves
• Curtains around beds
• Isolation of patients
*3. If a patient with TB is admitted - what action is taken and when?*
*4. Is action different when there is only suspicion of a TB?*
5. If the patient is transferred - are there any protective measures for suspected or confirmed cases?
*If yes, what are they? If no, why not?*
6. Which of these tasks in TB management do you find difficult to do?
• education on TB for patients and family- *If yes, why?*
• psycho-social counselling - *If yes, why?*
• training of family members - *If yes, why?*
• DOTS treatment - *If yes, why?*
• managing TB and co-infections - *If yes, why?*
• Caring for TB-patients in hospital - *If yes, why?*
• Supervision of health workers - *If yes, why?*
• TB-risk prone procedures - *If yes, why?*
• *Other tasks not mentioned - Which? Why?*
7. Are you afraid of acquiring TB at work? - *If yes, why, if no, why?*
8. Do you have concerns treating or working with TB patients? *If yes, why?*
9. Do you have concerns treating or working with TB patients with AIDS? *If yes, why?*
*10. How do you feel when these patients are on treatment for more than a week? Explain.*
11. Could you imagine referring a patient to a traditional healer for TB treatment, if requested by the patient? *Explain.*
12. Could you imagine referring a patient to a traditional healer as a therapist for DOTS, if requested by the patient? *Explain.*
13. Are there local beliefs that may influence TB-patients not to come to the hospital?
*If yes: what are they?*
14. Are there local beliefs that may influence TB-patients not to take TB-medication regularly?
*If yes: what are they?*
15. Did you participate in any TB-training or -workshop in the past two years? *If yes, what?*
16. Do you have sufficient access to information about TB-care, protective measures and other TB-related subjects to confidently manage patients? *If yes, from whom, how?*
17. Do you know the national TB-guidelines for health workers? *Explain.*
18. Do you know the policy for dealing with TB patients in your hospital? *Explain.*
19. Do you feel positive about the CPD-system (continuous professional development) that is already in place for doctors being applied for nursing staff as well in future? *Why?*

Choices of categories, if not otherwise indicated: yes/no. Open questions are marked in italic.

The interview guide was tested for content relevance and ease of application prior to data collection. Owing to a chronic staff shortage, compounded by a national health sector strike during the sampling period, a maximum of 20 nurses only were approved by the hospital management to participate in the study. Quota sampling was applied, including hospital wards where TB patients were managed on a routine basis (wards with "TB-routine", internal medicine, paediatrics and internal emergency wards), as well as wards where TB was not a clinical focus (wards "without TB routine", surgery, emergency trauma and obstetric). Nurses (n = 20) from the selected wards appearing on the off-duty plans during the sampling week were randomly selected and grouped into either working on such TB-routine wards (n = 10): emergency internal medicine (n = 2), paediatric (n = 4), internal medicine (n = 4); or not (n = 10): obstetric (n = 3), general surgery (n = 4) and emergency trauma (n = 3). The study included auxiliary nurses (AN) with one year training, staff nurses (SN) with two years training, and professional nurses (PN) with at least three years training. Nurses with different training curricula were included because due to staff shortage, all nursing levels were equally utilised for routine patient care.

### Data collection

The interviews, undertaken in private rooms offering strict confidentiality, were conducted in English by the Principal Investigator (PI), lasting approximately 30 minutes each. The participant responses were recorded in writing during the interview. Following the interviews, the PI immediately typed and cross-checked the coded scripts to ensure full and accurate data capture. Interviews were not audio-taped as the nurses felt that recordings could make them identifiable. This concern might have had a negative impact on their participation.

### Trustworthiness

The trustworthiness of the data was assured by testing the interview guide to identify and correct any ambiguities and/or errors, via one-to-one discussions with individual participants and peer reviewers, and by prolonged PI engagement in the field prior to and following data collection in order to achieve deeper understanding of the working context of the participants. The PI made every effort to clarify participants' responses and to verify the contextual appropriateness of the coding and emerging themes during data analysis. Two faculty members, and two IPC nurse specialists, served as peer reviewers to verify the thematic analysis, disagreements were debated to reach general agreement.

Throughout the research process the PI adhered to the importance of reflexivity [19], making explicit from the outset personal experiences, opinions and preconceptions about the field of research [20]. Throughout the research process, the PI employed "bracketing" [21] to suspend such personal perspectives and biases in order to reduce the phenomenon under study to its authentic basic components and actively searching for the fundamental concepts. The PI was an experienced clinical researcher who had spent time in the clinical IPC arena of TBH but came from a non-nursing background, thus providing professional working distance to the interviewees.

### Data analysis

The data was subjected to thematic analysis [22]. This method involved identifying, coding, analysing and clustering recurring factors into overarching themes with respective key and sub-themes. The identified themes are presented together with quotes from participants in order to add depth and richness to the findings. Participant quotations are used verbatim and presented in italics, and followed by a unique number indicated in brackets (#No.), to provide some context for the data [23] (Table 2).

**Table 2:** Nurses codes and characteristics (education, speciality, "TB routine")

Code #	Level of professional education	Speciality	TB routine* or not**
1	professional nurse	surgical	no
2	professional nurse	surgical	no
3	staff nurse	obstetric	no
4	professional nurse	obstetric	no
5	professional nurse	obstetric	no
6	auxiliary nurse	emergency trauma	no
7	professional nurse	emergency trauma	no
8	professional nurse	surgical	no
9	professional nurse	internal medicine	yes
10	auxiliary nurse	internal medicine	yes
11	staff nurse	internal medicine	yes
12	auxiliary nurse	internal medicine	yes
13	auxiliary nurse	internal medicine	yes
14	auxiliary nurse	internal medicine	yes
15	auxiliary nurse	pediatric	yes
16	staff nurse	pediatric	yes
17	professional nurse	pediatric	yes
18	staff nurse	pediatric	yes
19	professional nurse	surgical	no
20	staff nurse	emergency trauma	no

*TB routine: work in either internal medicine, paediatrics and emergency internal medicine

**no TB routine: work in either general surgery, obstetrics or emergency trauma

### Ethics

Ethical approval for the study was obtained from Stellenbosch University, and written consent was obtained from all participants. The interview scripts were coded, and personal identifying details were not collected.

## Results

The quota sample of 20 participants comprised PN (*n *= 9), (SN (*n *= 5), and AN (*n *= 6) (Table 2). All were female and of coloured ethnicity, their average age was 38 years and the mean working period in their current post was 10 years. Their mother tongue was Afrikaans but all were conversant in English. During the one-week sampling period, no males and nurses of African or white ethnicity were on the off-duty plan. Data saturation was reached within the sample.

The nurses expressed concerns about the possible risk of TB transmission to both patients and staff. Factors influencing TB-IPC, and increasing the potential risk of nosocomial transmission emerged in interconnected overarching themes, with respective key and sub-themes, related to the healthcare system, wider contextual conditions and patient behaviour (Table 3). Differences between experiences/practices of nurses working on wards with and without TB routine were noted, however, as participants were not representative, a direct comparison was not aspired.

**Table 3:** Interconnected contextual influences related to the healthcare system, wider contextual conditions and patient behavior on TB Infection Prevention and Control experiences of nurses (overarching themes, key themes & sub-themes)

OVERACHING THEMES	KEY THEMES	SUB-THEMES
**Influences related to the healthcare system**	IPC provision	*Isolation facilities*
		*Personal protective equipment*
		*TB policy*
	TB Training	*Nurses*
		*Patients/family members/carers*
	Communication	
	Work overload	
	Sense of duty of care	
**Influences related to wider contextual conditions**	TB concerns and stigma	*TB*
		*TB and HIV*
	Role of traditional healers	
**Influences related to patient behaviour**	Late uptake of hospital care	*Poverty*
		*Traditional medicine*
	Adherence to IPC measures by patients/family members/carers	

### 1. Influences related to the healthcare system

#### 1.1. IPC provision

The academic tertiary hospital had no designated TB wards, a closed ventilation system with only a limited number of rooms having access to natural ventilation, and only five rooms on one medical emergency ward were equipped with negative exhaust ventilation.

##### 1.1.1. Isolation facilities

The majority of participants from wards with TB-routine reported the availability of isolation rooms, curtains around beds, and general personal protective equipment (PPE) such as surgical masks and gloves. In contrast, almost all from wards without TB-routine, stated a lack of isolation facilities. **"***We don't have single rooms for isolation, patients have to lie amongst other patients. There is continuous lack of space. Also, only half of the rooms are equipped with curtains. Masks are not practical. At the end, patients can stay here for one to two days before they are sorted out and the staff is not protected" *(#4).

All participants from wards without TB-routine were of the opinion that their working environment was not equipped to prevent TB transmission. They explained that although patients were not admitted to the ward for very long, the risk of TB exposure was high due to the lack of isolation facilities, failure in provision of PPE, and a high turn-over of patients. Even on the one ward with designated isolation facilities, patients with infectious TB would not be isolated because doctors focus primarily on the surgical conditions of patients.

Furthermore, most of the participants from wards with TB routine admitted that periodically their isolation rooms were used for immuno-suppressed patients instead of those with infectious TB. "*We keep the TB-patients in the front in order to protect the haematological patients in the back*" (#10). Gender considerations also hampered respiratory isolation practices since male and female patients could not be mixed at ward level. Based on the findings, isolation was performed on a regular and consistent basis only on one internal medical ward, indicating that the risk of nosocomial transmission was high on all other wards.

##### 1.1.2. Personal protective equipment

If fitted and used properly to prevent face seal leaks, a respirator can greatly reduce the chance that inhaled air will contain infectious tubercle bacilli [5]. Even a surgical mask that filters only 50% of inhaled particles has the equivalent effect, in theory, of a doubling of room ventilation, and at a fraction of the cost [5]. Although surgical masks, as part of standard PPE at the hospital, were available on most wards, some participants from wards without TB-routine admitted that these were not used at all for this purpose. Most participants indicated that there was a lack of knowledge about the appropriate use of masks and respirators. N95-respirators were available on most of the wards with TB-routine and, in line with local recommendation, only used in the case of drug-resistant cases. Those from wards without TB-routine reported an absence of N-95 respirators.

##### 1.1.3. TB policy

The participants were unanimous in their view that clear directives, including standard operating procedures and standards of care, were missing and contributed to inconsistencies in practices. None of the participants knew about the SA National TB guidelines [24]. Some knew about a TB policy at the hospital but were unaware of the content. A hospital TB policy existed in an English draft version only and seemingly had not been made known to all ward nurses. Triaging symptomatic patients to expedite care and reduce the amount of potential exposure to others, is recommended in a draft national policy [25]. On the wards, only a small number of nurses routinely assessed newly admitted patients for symptoms and signs of TB. The majority of participants did not use their own clinical judgement concerning the need for TB-triage. They relied on physicians' instructions as "...*it is not part of the ward plan*" (#1) or "...*there is no time*" (#6) to undertake a TB risk assessment. Accordingly, most participants, including those on wards with TB-routine stated that patients with suspected TB were not subjected to IPC measures like the use of transmission-based precautions. "*No action is taken until the results come back. Then treatment and education of how to cough is started. There is no isolation*" (#5).

Only half of participants from wards without TB-routine, and almost all from wards with TB-routine, reported the provision of surgical masks to patients confirmed with TB when transferred to another ward. The appropriate IPC measures were applied by most nurses only if patients had MDR-TB. The remaining participants did not apply any transmission-based precautions when transferring patients with TB. "*Usually we inform the ward of the diagnosis tests that are done. We don't use masks. The patients take the same lifts, the same passages like any other people in the hospital*" (#17).

#### 1.2. TB training

A recurring theme was the deficit in TB-related training.

##### 1.2.1. Nurses

None of the participants had received any training in the previous year, and none had undertaken any TB-IPC courses. Participant responses exhibited misconceptions and lack of knowledge about the time period during which patients remain infectious following the commencement of treatment. Wrongly believing that the patient is immediately rendered non-infectious on commencement of treatment, some participants, a few from wards with TB-routine, immediately stopped applying the appropriate IPC measures (transmission-based precautions) "...*once the first pills are swallowed*" (#14). Others did not trust the effect of treatment and continued transmission-based precautions for the entire period of patient admission.

All participants urged implementation of a continuous professional development (CPD) system for nurses, currently only available for doctors in SA. "*Nurses must stay up to date with the current knowledge and latest technologies. There are colleagues who have been working for twenty years with no knowledge to deal with new developments*" (#2).

##### 1.2.2. Patients/family members/carers

However, not only health staff but also patients, their family members and carers, desperately needed knowledge about TB: "*Some don't know what TB is about, that TB can be cured and how TB is spread. There are myths and stigma attached to it" *(#17). The participants were unanimous in their views that such knowledge would support patient's adherence to IPC measures, facilitate the treatment and care process, and motivate family members and carers to be screened for TB.

#### 1.3. Communication

Participants felt strongly that cultural and linguistic differences between staff and patients created communication barriers in clinical IPC practices: *"Sometimes it is a language problem, sometimes I don't understand patients' motives" *(#19). There was a general consensus that undiagnosed TB-patients, many of them being of African origin, experienced communication constraints in revealing possible infection when admitted, and, in the course of hospital admission, experienced difficulties in understanding the TB diagnosis and treatment procedures: "*We give advice but we are not sure if they understand*" (#6).

#### 1.4. Work overload

The majority of participants from wards without TB-routine reported that TB-related duties were often being neglected owing to work overload which was exacerbated by a chronic staff shortage and competing clinical priorities. *"TB is not our main concern. We are always in a hurry to get our work done. There is no time to chat with our patients" *(#5). The responses from participants also reveal the impact of work overload on the hospital discharge of patients. *"Usually we give tablets and they are referred to the ID clinic. We neglect to explain more thoroughly. Not all patients go there, they return to their community without a chance to get a proper explanation" *(#19).

#### 1.5. Sense of duty of care

A view that featured prominently was concern about the potential transmission of TB to patients due to the lack of IPC measures in place: *"We try to prevent cross-infection to other patients, but often they are not separated. With six patients in one room, distance is not far. Some have nasty sputum and produce a lot of it. I tell them to control the stuff and not to pile it up in regards to other patients" *(#19). Despite the range of contextual influences impeding TB-IPC at ward level and posing a risk for their own health, all the participants expressed a strong sense of duty of care: "*We have a lot of sick people, they need us. If they didn't have us - what would these people do?*" (#10).

### 2. Influences related to wider contextual conditions

#### 2.1. TB concerns and stigma

The participants revealed the existence and impact of concerns and stigma about TB. There is considerable secrecy and stigmatisation attached to this disease [11].

##### 2.1.1. TB

Most participants, all among those from wards without TB-routine, expressed concerns about becoming infected with and developing TB disease. Stated reasons for concern were: childcare responsibilities at home, prolonged treatment and side effects, and workplace and social stigma associated with TB. Despite these reported personal concerns, most participants did not express fear about working with TB patients. One stated a healthy lifestyle as a reason for not being afraid of acquiring TB at work: "*I eat healthy, there are many diseases in the hospital, but my own body is strong*" (#9).

##### 2.1.2. TB and HIV

The majority of participants reported no concerns in nursing TB patients co-infected with HIV. Their reported fears related to TB rather than HIV, attributed to better training received in HIV than TB care. Only one nurse worried about iatrogenic HIV infection: "*I wouldn't want to inject HIV patients as I am worried about needle pricks" *(#8).

In contradiction, when discussing patients' possible concerns, most participants felt that patients were more worried about HIV than TB: *"There is more stigma against HIV than against TB. TB is not so much a problem because it can be treated. The stigma makes people distant themselves from patients with HIV as soon as they know. People are afraid to get it, they are not educated about it" *(#13). Several nurses were of the opinion that some patients with TB symptoms delay accessing medical care owing to concerns about being diagnosed with HIV. *"Patients are too ashamed or scared to come to the hospital as they know that TB and HIV are linked. They are worried about the diseases and consider their personal and family situation. They would have to bring the family to the hospital as well and then they have to reveal their status. Therefore, instead, they go to traditional healers. They think they can solve their problem without tablets" *(#7).

#### 2.2. Role of traditional healers

All participants were ambivalent and concerned about the role of traditional healers in TB care. Some felt, that healers were often the first point of care for many patients to *"...solve their TB problem" *(#7) since they were promised contact with their ancestors to enquire about the best treatment option and/or a one-week curative treatment. "*Traditional healers are paid a lot of money because some of the patients have belief that if they speak with ancestors they are given the knowledge to treat TB" *(#5).

None of the participants could imagine referring a TB patient to traditional healers for treatment, most felt that this would deteriorate the health of the patient. *"One patient brought a bottle of stinking stuff. She drank it and vomited afterwards. There is no cure with them" *(#4).

However, if traditional healers worked "hand-in-hand" with medical staff and were requested by the patient, some were prepared to refer for directly observed therapy short course (DOTS) treatment support because "...*they are often an authority for the patients that medical people are not*" (#20). The importance of providing TB and DOTS health education to traditional healers before referring patients was stressed by all.

### 3. Influences related to patient behaviour

#### 3.1. Late uptake of hospital care

Late uptake of hospital care by adult patients, and guardians of paediatric cases, was a recurring concern. Such delays were attributed to several influences.

##### 3.1.1. Poverty

Poverty, in particular a lack of money for transport, was perceived as the principles cause for the late uptake of hospital care: *"Patients come from poor conditions and live far from the hospital. Some of them don't have proper housing, they stay in shacks, their nutritional status is bad, they have no work and no money to go to the hospital" *(#16).

Participants working in paediatric settings explained that poor parents of children with TB symptoms would be worried to reveal personal impoverished living conditions to hospital staff in case their children are taken into custody. Consequently, they delay the uptake of medical care.

##### 3.1.2. Traditional medicine

Many participants expressed strong opinion that patients accessed medical TB care only after trying traditional treatment and when physically extremely unwell, or for another medical condition. "*Some patients only come if they are really sick and see that herbal medicine doesn't help*" (#5).

#### 3.2. Adherence to IPC measures by patients/family members/carers

Most participants from wards with TB-routine explained that patients with pulmonary TB (PTB) repeatedly failed to follow IPC measures, such as cough etiquette and mask usage: *"There is a lack of understanding between the patient and the nurse. Most of the patients are stubborn and their behavior is uncooperative. They must lie here for weeks without any test being done any more. They could get their treatment outside. They have a bad attitude, don't want to eat, carelessly cough around, refuse their tablets. They are a risk for everyone. Often I don't trust them" *(#12). *"Especially MDR patients cough around, keep their room untidy and some of them refuse to take their tablets*" (#20). The reported lack of adherence also applied to the family members and carers of TB patients who sometimes *"refuse to go for a test or X-Ray. Some have symptoms, but they are afraid to accept the truth" *(#6).

A participant from a paediatric ward complained that often only one parent accompanied a child to the hospital and after discharge would not properly inform the other parent about the required TB care. In consequence, children would often have to be readmitted, sometimes with drug resistant TB.

The majority of participants described several perceived reasons why TB patients might default on their medication. Side-effects were cited most often. *"Sometimes, tablets are too much, often patients don't have food to eat and their medicine has side effects" *(#7). Discolouration of the urine by rifampicin could also cause non-adherence to treatment if patients were not properly educated at the start of treatment. The misuse of medication was named as a further reason for defaulting. "*Some adolescence steel antiretrovirals and TB-drugs to inhale them as narcotics*" (#9).

The influence of poverty in this context was also mentioned. "*In poor communities, there is awareness that alcohol and drugs might interfere with treatment. That's why they don't take their drugs" (#*3). It was also explained that a social grant, established for poor and moribund TB-patients, could encourage non-adherence with treatment because cure of TB resulted in termination of the financial allowance. One participant reported a strategy used by a patient to qualify for the grant: "*A patient presented with haemoptysis and the doctor had the blood analysed. It was found to be sheep blood*." (#13).

## Discussion

The study has obtained the experiences of ward nurses concerning multiple factors influencing TB-IPC practices and increasing the potential risk of nosocomial transmission at hospital level. The key perceived contextual influences fall into broad interconnected overarching themes related to the healthcare system, wider contextual conditions and patient behaviour. These broad themes correspond with those elicited in a study exploring barriers to TB care in Russia [14].

The major influences were inadequacies associated with the healthcare system. Routine IPC audits at different departments of our hospital had showed inconsistently applied transmission-based precautions, even where known TB cases were admitted on a routine basis [7]. Effective TB-IPC practices by nurses were hampered by the lack of clear TB policy directives, the lack of appropriate isolation facilities and availability of PPE, the lack of TB training for staff and patients, and a persistent work overload.

The findings illuminate the need for a comprehensive TB-IPC policy, with associated standards for provision and practice. The infection control policy must reflect current guidelines on infection control, as well as legislative issues and evidence of annual audits thereof [26]. It should also be in line with the International Standards for TB Care [27]. Healthcare workers cannot be blamed for applying inconsistent TB-IPC measures when appropriate, accessible and clear guidelines are not implemented, monitored and evaluated. According to our experiences, and in line with internationally agreed standards, IPC practices should include: rapid clinical evaluation (TB-triage) of all persons (staff, patients and visitors) with symptoms suggestive of TB, segregation of patients with known or suspected pulmonary TB, use of effective local exhaust ventilation in connection with high-risk procedures, employee training, and ongoing risk assessment [28]. Especially participants from wards without TB-routine complained that patients were managed inadequately with regard to TB. There was no consistent TB-triage by nurses on patient admission, and suspected TB cases were not included in TB-IPC measures such as isolation and usage of PPE. On transfer, only patients with MDR-TB were usually provided with masks. Other PTB patients, confirmed or suspected, were dealt with by chance, dependant on individual staff practices. A large hospital without basic measures for airborne IPC, combined with the high HIV infection prevalence among patients and visitors, could provide a prime setting for the rapid spread of TB.

Work practices and administrative control measures are the first line of defence against TB transmission within facilities caring for people with HIV infection [5]. A previous study reported that at the hospital 54% (107/199) of TB patients were HIV-positive and one fifth of PTB patients were diagnosed on wards without TB-routine [7]. HIV infected persons must be protected from exposure to TB as far as possible, with special care needed in healthcare facilities, especially hospitals. TB suspects and diagnosed patients should be managed in separate places and environmental controls including good ventilation must be in place [26]. The findings of the study suggest that the hospital was not adequately equipped to protect HIV positive patients from acquiring TB transmission, especially on wards without TB - routine. Patients were segregated according to their clinical profile and gender, not to their TB status.

Similar to the study by Dimitrova et al. [14], the findings reveal staff shortage as a significant problem, leading to work overload, competing clinical priorities and working in a high risk environment. Despite such negative working conditions, the participants expressed a strong sense of duty for care.

All healthcare staff, including support staff, must receive training in infection control [29]. Through ongoing training and CPD, nurses could develop the skills and enhance their confidence in TB and HIV care in a high burden environment and make informed clinical judgements on appropriate management. The findings of the study identify the pressing need for TB training for all nurses. The participants did not seem fully to comprehend the real risk of TB exposure, and most were afraid of becoming infected with and developing TB. Concerns and stigma related to TB emerged particularly among participants from wards without TB-routine. Irrational work practices and fear might lead to a higher risk of exposure and nosocomial transmission.

IPC training interventions should urgently be offered for nurses and other healthcare workers to improve knowledge, clinical competency and quality of care.

Concerns and stigma related to TB and HIV, and the role of traditional healers, comprised wider contextual influences which impeded TB-IPC at ward level. At the time of the study, approximately 80% of nurses at the hospital spoke Afrikaans as first language, and three quarters were of coloured ethnicity, followed by an equal proportion of African and white nurses. A previous study at the hospital found that about half of the patients with PTB were of African origin, the other mainly coloured [7]. In the catchment area of this hospital, the African population predominantly spoke Xhosa as first language. Most of the study participants who reported problems concerning cross-cultural understanding of stigma and communication, originated from a different cultural background. The findings suggest a deficit in knowledge of cross-cultural health beliefs. Similar observations were reported in the Limpopo Province of SA [11].

The impact of traditional healers on patient health was raised by participants. Patients with TB, especially those from an African cultural background, would commonly seek a first opinion from traditional healers, resulting in the late uptake of hospital care. A South African study reported that 74% of patients with TB had visited a traditional healer before attending hospital, and that patients who visited traditional healers took longer to access anti-tuberculosis chemotherapy, were in worse condition by the time they presented, and were more likely to die after they had presented with TB [11]. The strength and depth of the cultural links between traditional healers and their local communities suggest that such authorities cannot simply be ignored or chastised [30-32]. The involvement of traditional healers in treating TB patients was rejected by the study participants, suggesting a general poor opinion about and understanding of the role of traditional medicine. Only traditional healers trained in TB therapy support were acceptable to some participants.

Although there is strong evidence that the accessibility and acceptability of health services remain the most important factors in patient adherence [33], patients behaviour is also of critical importance in TB-IPC. Patients' late presentation to hospital was reported in other SA studies [34,35]. While the authors saw this phenomenon mainly caused by health system failure, Dimitrova et al. [14] identified fear of unemployment and stigma towards TB as a major obstacle. In our study, most nurses linked patients' late presentation to hospital mainly to the impact of poverty and traditional medicine. In the study by Dimitrova et al [14], the 'willingness' to access and adhere to treatment was seen as a critical factor in successful treatment outcomes. In our study, side effects related to TB treatment, patients' concerns and stigma related to HIV and the lack of TB knowledge of patients and their family members/carers were seen as major influences on patient adherence.

In reviewing the role of TB education for patients, Sumartojo [36] emphasized the need to facilitate behavioural change rather than simply to provide disease information. Edginton et al. [35] found, that although non-attending patients were visited at their homes by researchers who told them about TB and the need for treatment, more than one third still did not attend. This indicates the complexity of contextual influences on decision making. In a local context of multiple cultures, ethnicities and languages, highly specific and culturally sensitive educational interventions are required and should be offered both to patients and their family members/carers in their mother tongue. Acknowledging the role of traditional medicine, consideration should be given to involving traditional healers in such TB educational interventions to facilitate early medical care, treatment support and continuity of care, and to improve TB-IPC at both community and hospital level.

### Strengths and Limitations

TB nurses perform the bulk of work in TB care and control [37]. This study recognises their crucial role and emphasizes the importance of well-trained staff. A focus group with nursing participants to explore in more depth and/or illuminate further contextual influences on TB-IPC, and to serve as a form of triangulation, was not approved by the hospital management owing to staff shortage. Similar to other qualitative studies, this study was context-specific, hence the findings cannot be extrapolated to other settings. The restricted sampling period resulted in failure to include males and nurses from African ethnicity in the random sample. Their inclusion might have yielded additional insights. It would have been invaluable to explore the cultural context of African patients from the perspectives of African nurses. The staff crisis at the time of sampling also lead to limited transferability, as only motivated nurses and staff being able to deal with work overload were accessible. Nevertheless, this study highlights several issues of critical importance in TB-IPC as experienced by those in the frontline of care provision. The findings might reflect the realities and influences experienced by many nurses in other settings, and pose implications which could be considered for TB-IPC policy and practice in similar settings elsewhere.

## Conclusion

Healthcare system inadequacy was the major influence on TB-IPC. IPC provision and practices, TB training for staff and patients, and cross-cultural communication were perceived by nurses to be suboptimal at this large hospital and could increase the risk of nosocomial transmission. The first step should be the implementation and evaluation of a comprehensive contextually appropriate TB-IPC policy with the setting and auditing of standards for appropriate IPC provision and practice across all wards. Concerns and stigma attached to TB should be addressed among both healthcare providers and patients. Cross-cultural and TB training for nurses, other healthcare staff including executive managers, and patients, should be a priority in high TB-burden hospitals. Urgent attention should be given to the establishment of a cross-cultural communication strategy, including rapid access to interpreters.

## Competing interests

The authors declare that they have no competing interests.

## Authors' contributions

DS conducted the in-depth interviews. All the authors have (1) made substantial contributions to study conception and design, (2) been engaged in data analysis and interpretation of data, (3) been involved in drafting the manuscript or revising it critically for important intellectual content; and (4) have given final approval of the version to be published.

## Pre-publication history

The pre-publication history for this paper can be accessed here:

http://www.biomedcentral.com/1471-2458/11/262/prepub
